# Spectrum of Pituitary disorders: A retrospective study from Basrah, Iraq

**DOI:** 10.12688/f1000research.13632.2

**Published:** 2018-06-22

**Authors:** Abbas Ali Mansour, Ali Hussain Ali Alhamza, Ammar Mohammed Saeed Abdullah Almomin, Ibrahim Abbood Zaboon, Nassar Taha Yaseen Alibrahim, Rudha Naser Hussein, Muayad Baheer Kadhim, Haider Ayad Yassin Alidrisi, Hussein Ali Nwayyir, Adel Gassab Mohammed, Dheyaa Kadhim Al-Waeli, Ibrahim Hani Hussein

**Affiliations:** 1 Faiha Specialized Diabetes, Endocrine and Metabolism Center (FDEMC), Diabetes, Endocrine and Metabolism Division, Department of Medicine, Basrah College of Medicine,Hattin post office. P.O Box: 142, Basrah, 61013, Iraq

**Keywords:** Sellar and parasellar region lesions, pituitary disease, pituitary adenoma, classification, epidemiology.

## Abstract

**Background:** Pituitary disorders spectrum includes a wide variety of diseases.This study aimed at a comprehensive description of such disorders for patients from  Faiha Specialized Diabetes, Endocrine and Metabolism Center (FDEMC) in Basrah (Southern Iraq).

**Methods: **Retrospective data analysis of FDEMC for the period from January 2012 through June 2017. We included all patients with pituitary disorders who have MRI pituitary.

**Results: **The pituitary disorders were more common among women. Those with macroadenoma were older than those with microadenoma with nearly equal gender prevalence of macroadenoma. Pituitary adenoma constituted the bulk of pituitary disorders in this registry (67.2%). Growth hormone secreting adenoma were the commonest adenoma seen in 41.0% followed by clinically non-functioning pituitary adenoma(NFPA)in 31.4% and prolactinoma in 26.9%. About 64.8% of pituitary adenoma was macroadenoma. Macroadenoma was seen in 73.4 % of growth hormone secreting adenoma, 61.2% in NFPA and 62.0% of prolactinoma (of them six were giant prolactinoma)

**Conclusion**: Pituitary adenoma constituted the bulk of pituitary disorders in Basrah, growth hormone secreting adenoma is the commonest adenoma followed by NFPA and prolactinoma due to referral bias. A change  in  practice of pituitary adenoma treatment is needed.

## Introduction

Sellar and parasellar region lesions spectrum includes a wide variety of conditions ranging from adenoma to empty sella syndrome, apoplexy, congenital or acquired condition ^1– 4^. Other than adenoma, genetic causes of pituitary disease are increasingly recognized ^3^.

Pituitary adenomas are not rare and account for 20% all intracranial tumors ^5, 6^. Half of these secrete hormones, and half are microadenoma ^2^. Clinically non-functioning adenomas (NFPA) constitute 15–54% of all adenomas. Prolactinomas accounts for 32–66%, growth hormone secreting adenoma (acromegaly) account for 8–16%, adrenocorticotropic hormone (ACTH)-secreting adenoma (Cushing's disease) forms 2–6%, and TSHoma accounts for less than 1% ^2, 7^. These pituitary adenomas behave as typical or have a more aggressive to malignant behavior ^6, 8^. They can cause mass effect, in addition to hypersecretion or hypopituitarism ^7, 9^.

Advances in neuroradiology have opened the door for earlier and easier diagnosis of pituitary disease and other sellar and suprasellar lesions ^10^.

The Faiha Specialized Diabetes, Endocrine, and Metabolism Center (FDEMC) in Basrah is a tertiary referral center receiving patients with pituitary diseases from most of Southern Iraq. The FDEMC is trying to adapt the three mission criteria of the pituitary center of excellence, which includes care and support for patients, fellowship training and contribution to pituitary disease research ^11^. To our knowledge, there are no studies on sellar and parasellar region lesions in Iraq.

This study aimed at a comprehensive description of pituitary disorders for patients from FDEMC in Basrah (Southern Iraq).

## Methods

### Study design

Retrospective data analysis of FDEMC database for the period from January 2012 through June 2017.

**Inclusion criteria**: We included all patients with pituitary disorders who have MRI pitutiary regardles the age.

**Exclusion criteria**: patients with pituitary disorders with missed MRI.

## Definition of variables

Sequences of pituitary MRI imaging were classified according to the international standard ^12^. Adenomas were classified as macroadenoma if these were 10 mm or more in size, while microadenoma if less than 10 mm and giant prolactinoma if these were 4 cm and above ^2^.

Pituitary adenoma (NFPA, prolactinoma, growth hormone secreting adenoma [acromegaly], and adrenocorticotropic hormone (ACTH)-secreting adenoma) were defined according to the usual criteria ^2, 8, 12^.

Hypopituitarism, whether postoperative or in those with or without adenoma, was considered according to the hormonal assessment with basal and dynamic hormonal tests ^13^.

Empty sella syndrome, whether primary or secondary to surgery or apoplexy, were considered based on MRI findings ^14^.

Craniopharyngioma diagnosis was based on clinical behavior with MRI and pathological diagnosis.

## Data analysis

Analysis was done in July 2017. All patients with labeling diagnosis of pituitary disease were included. Data were included on an Excel spreadsheet and transferred to SPSS for Windows, Version 23.0 (SPSS Inc., Chicago, USA).

Continuous variables were summarized as number and percentage and dichotomous variables as mean ±SD.

### Ethics statement

The ethics committee of the Medical College in Basrah University approved the study design and the Center authorities agreed to review the patients data. At the time of registeration in the Center, all patients included in this study approved the use of their clinical information for research purposes.

## Results

A total of 232 patients were included in this study. Pituitary disorders were more common among women ( Table 1). Those with macroadenoma were older than those with microadenoma with nearly equal gender prevalence of macroadenoma. Four patients died; two with growth hormone secreting adenoma (acromegaly) and advanced cardiovascular disease, and two with prolactinoma that caused hypopituitarism and adrenal failure.

**Table 1.** Pituitary disorders patients demography and characteristics.

			N (%); mean ±SD
Gender	All pituitary disorders	Men	84 (36.2)
		Women	148 (63.8)
	Pituitary adenoma *	Men	67 (43)
		Women	89 (57)
Age at registration, years		All	38.2±15.3
		Macroadenoma	42.5±14.9
		Microadenoma	34.8±14.7
Macroadenoma **		Men	51 (50.5)
		Women	50 (49.5)
Died			4

*Of 156 pituitary adenoma **Of 101 macroadenoma

Table 2 shows that pituitary adenoma constituted the bulk of pituitary disorders in this registry (67.2%). Growth hormone secreting adenoma (acromegaly) were the commonest adenoma seen in 41.0% followed by NFPA in 31.4% than prolactinoma in 26.9%. Hypopituitarism due to various causes was observed in 24.5% in this series. Empty sella syndrome, whether primary or secondary, were seen in 9.4%. Craniopharyngioma and Sheehan syndrome were seen in 3.9% each. Meningioma based on MRI finding was been observed in 4 patients (1.7%).

**Table 2.** Spectrum of pituitary disorders at the time of registry.

		N (%)
Pituitary adenoma		156 (67.2)
Growth hormone secreting adenoma (acromegaly)		64 (41.0)
Clinically non-functioning pituitary adenoma (NFPA)		49 (31.4)
Prolactinoma *		42 (26.9)
GH-secreting adenoma with hyperprolactinemia *		5
ACTH- secreting pituitary adenoma		2 (1.2)
Hypopituitarism		57 (24.5)
Empty sella syndrome	All	22 (9.4)
	Primary **	*9*
	*Secondary*	*13*
Diabetes insipidus		15
Apoplexy		3
Hyperprolactinemia	All	51
	No adenoma	12
Pituitary enlargement		3
Stalk lesions		1
Miscellaneous		***11
Craniopharyngioma		9 (3.9)
Sheehan syndrome		9 (3.9)
Meningioma		4 (1.7)
Rathke's cleft cyst		3
**Total**		**232**

*GH-secreting adenoma,2 of them stain on biopsy for lactotroph cell **Acromegaly in 4 *** Miscellaneous includes galactorrhea, hypogonadotropic hypogonadism, and acromegaloidism

In this study, 64.8% of pituitary adenoma were macroadenomas ( Table 3). Macroadenoma was seen in 73.4% of acromegaly, 61.2% in NFPA and 62.0% of prolactinoma (of them six were giant prolactinoma).

**Table 3.** Pituitary adenoma according to the size.

	Microadenoma N (%)	Macroadenoma N (%)	Total
Pituitary adenoma	55 (35.2)	101 (64.8)	156
Growth hormone secreting adenoma (acromegaly)	17 (26.5)	47 (73.4)	64
Clinically non- functioning pituitary adenoma (NFPA)	19 (38.8)	30(61.2)	49
Prolactinoma	16 (38)	26 (62.0) *	42
ACTH- secreting Pituitary adenoma	2 (100.0)	0	2

*Of them six giant Prolactinoma

In Table 4 we see hypophysectomy whether transsphenoidal or transcranial or both was performed in 45 patients with pituitary adenoma (28.8%). Stereotactic radiosurgery is done in 5 patients (3.2%) with pituitary adenoma. Growth hormone secreting adenoma (acromegaly) and prolactinomas were treated primarily with medical therapy (71.4% and 76.1% respectively).

**Table 4.** Type of treatment for pituitary adenoma.

		N (%)
Hypophysectomy-transsphenoidal		33 (21.1)
Hypophysectomy-transcranial		8 (5.1)
Hypophysectomy-transsphenoidal followed by transcranial or reverse or repeat same surgery,i.e., twice surgery		4 (2.5)
Stereotactic radiosurgery		5 (3.2)
Radiotherapy		1 (0.6)
Primary medical treatment	Growth hormone secreting adenoma (acromegaly)	46 (71.4) *
	Prolactinoma	32 (76.1) **
Total		156

*Of patients with acromegaly **Of patients with prolactinoma

Description of patients included in the study Click here for additional data file. Copyright: © 2018 Mansour AA et al. 2018 Data associated with the article are available under the terms of the Creative Commons Zero "No rights reserved" data waiver (CC0 1.0 Public domain dedication).

## Discussion

All pituitary disorders and adenoma were more common among women in this study. The gender predominance among patients with pituitary adenoma is variable in the literature depending on hormone secretion and age of the patients, the size of the tumor and female dominance is not clear ^15, 16^. However, female dominance has been seen in Saudi Arabia ^17^ and one series from Argentina ^18^. Those with macroadenoma tend to be older in age with no difference in the prevalence between men or women.

Seen in about two-thirds of patients, pituitary adenoma constituted the main bulk of pituitary disease in this study, which is compatible with reports in the literature ^16^.

The commonest pituitary adenoma was growth hormone secreting adenoma (acromegaly), followed by NFPA and prolactinoma. This is entirely different from the literature on the prevalence of pituitary adenoma ^2, 16, 17, 19^. This could be attributed to selection bias because only growth hormone secreting adenoma (acromegaly) patients are being referred, while NFPA and prolactinoma were treated by different specialties, such as neurosurgeons or gynecologists, without referral to a specialized Center like FDEMC. In Basrah, most cases of hyperprolactinemia were seen by a gynecologist because of amenorrhea and infertility, and the neurosurgeon follows patients with NFPA without referring them.

Hypopituitarism is prevalent in a quarter of this pituitary centre, from different causes, ranging from macroadenoma to hypophysectomy. Evaluation for hypopituitarism remains an integral part of the workup for any pituitary lesions because missing such diagnosis could be catastrophic ^9, 13^. This figure is far higher than that of Saudi Arabia, which was 1.2% ^17^.

Empty sella syndrome was seen in 9.4% of patients in this study, which can be primary or secondary to surgery or apoplexy. Empty sella syndrome needs an extensive workup to assess pituitary function ^20^.

Craniopharyngioma and Sheehan syndrome are two diseases with a different spectrum of age distribution, but they were seen at the same frequency in this cohort. Craniopharyngioma is a disease of childhood and adolescence ^21^. Sheehan syndrome is supposed to be rare in developed countries, but is still seen in developing countries ^22^.

Less than two thirds of adenoma in this study were macroadenomas. While in most series macroadenomas constitute 50% of the pituitary adenomas ^2^; however in Canada, a similar finding has been seen compared with this study ^23^. Again this could be explained by referral bias in this study. In Saudi Arabia, microadenomas were more prevalent ^17^.

For growth hormone secreting adenoma (acromegaly), more than two thirds were macroadenomas, which is established fact for all acromegaly ^2, 24, 25^.

NFPA was a macroadenoma and seen at around 60% in this study. A similar finding was seen in a previous series ^2^.

Prolactinomas were macroadenoma in around 60% of cases in this study. This differs from the literature, where more than 90% of prolactinomas were microadenomas ^2, 18^.

Hypophysectomy-transsphenoidal as surgical treatment was done in one third of pituitary adenomas, while transcranial approach or stereotactic radiosurgery was contemplated in the minority. This is a typical approach for most of the pituitary adenomas ^2, 26^. For growth hormone secreting adenoma (acromegaly), the primary treatment in this study was medical treatment in about two thirds of individuals. This is contrary to literature where surgery is the main mode of therapy ^26^. The explanation is that we are just building a new neurosurgery unit for pituitary glands over the last few years, and in the future, surgery of pituitary is supposed to improve, and early referral will be the best.

For prolactinoma, primary medical treatment was done in two thirds of patients, while it should be the main treatment of choice in more than 90%, as seen in previous literature ^26^.

Malignant disease metastasizing to the pituitary is not observed in this study because they are not referred from Oncology Center in Basrah.

### Study limitation

This study supposes to involve most of the pituitary disease patients in Basrah because the Center is a tertiary referral center. However, due to referral bias among some neurosurgeons and gynecologologists, we cannot guarantee that the data includes all patients with this condition in Basrah.

## Conclusion

Pituitary adenomas constituted the bulk of pituitary disorders in Basrah. Growth hormone secreting adenoma (acromegaly) is the most frequent adenoma followed by NFPA and prolactinoma due to referral bias. A change in the practice of pituitary adenoma treatment is needed.

## Data availability

The data referenced by this article are under copyright with the following copyright statement: Copyright: © 2018 Mansour AA et al.

Data associated with the article are available under the terms of the Creative Commons Zero "No rights reserved" data waiver (CC0 1.0 Public domain dedication). 

**Dataset 1:** Description of patients included in the study 10.5256/f1000research.13632.d197439 ^27^
