# Supplementary material for: Spectrum of Pituitary disorders: A retrospective study from Basrah, Iraq
Source: F1000Res. 2018 Jun 22;7:430. Originally published 2018 Apr 6. [Version 2] doi: 10.12688/f1000research.13632.2 (PMC6039954; doi:10.12688/f1000research.13632.2)
Supplement: Peer review history

# Peer review: Referee response for version 2

**DOI:** 10.5256/f1000research.16733.r35342

**Published:** 2018-07-09

**Authors:** Khaled Mohammed Al-Dahmani

**Affiliations:** 1 Divison of Endocrinology, Tawam Hospital, Al Ain, United Arab Emirates; 2 Department of Medicine, College of Medicine and Health Sciences, United Arab Emirates University, Abu Dhabi, United Arab Emirates

While the authors addressed some of the raised comments, I see a need for the following:

1. Simplify Table 2 into 4 main groups as mentioned before. It is understandable that patients with seller masses may also have hyperprolactinemia and hypopituitarism. These cases should be counted  under sellar masses category and not included under hyperprolactinemia and hypopituitarism catergories. This will clarify the spectrum of pituitary disorders seen in this study and will be easy to compare if similar studies from other centers were to be published in the future.
2. Under ''miscellaneous'' group, galactorrhea and acromegaloidism need not to be included in the study unless there is a pituitary disorder to explain such diagnosis. The other case(s) of hypogonadism should be counted under the category of hypopituitarism  (which is already present as a category by itself).
3. For Table 3, the total number of adenomas (156) should exactly equal the sum of all adenoma subtypes. If a patient has a mixed adenoma (GH & PRL secreting adenoma) it should be counted as 1 adenoma under GH category for example and not included both.

I have read this submission. I believe that I have an appropriate level of expertise to confirm that it is of an acceptable scientific standard.

# Peer review: Referee response for version 1

**DOI:** 10.5256/f1000research.14810.r34531

**Published:** 2018-06-14

**Authors:** Khaled Mohammed Al-Dahmani

**Affiliations:** 1 Divison of Endocrinology, Tawam Hospital, Al Ain, United Arab Emirates; 2 Department of Medicine, College of Medicine and Health Sciences, United Arab Emirates University, Abu Dhabi, United Arab Emirates

The study by Mansour AA et al. is an important contribution to the limited pituitary research in the middle East and North Africa (MENA) region.

 The following points need further modifications/clarifications;

- Sellar and parasellar region lesions usually refers to structural abnormalities and do not necessarily include other pituitary pathologies like hyperprolactinemia and hypopituitarism. Therefore, I suggest using the term ''pituitary disorders'' instead; more comprehensive.
- The inclusion of patients needs further clarifications in the ''Method'' section. what diagnoses where looked for?
- Pituitary adenoma includes all functioning and non-functioning pituitary tumors. In some part of the text, it was used separately from its subtype (see paragraph; Definition of variables). Maybe just typo. 
- Table (2) needs to be simplified. The total number of patients should equal clearly equal 232. I suggest using 3-4 categories only; Sellar masses/abnormalities, Hyperprolactinemia, hypopituitarism and others.
- The "miscellaneous" cases need to be mentioned; what diagnoses are here?
- Table 3 the sum of patient with pituitary adenoma subtypes are not equal to the  total number of cases.
- In the conclusion, ''A change in the practice of adenoma treatment is needed'' is very general. Based on the presented data, change in the management of GH secreting adenomas is more specific and relevant.
- Additional language editing will further enhance the manuscript.

I have read this submission. I believe that I have an appropriate level of expertise to confirm that it is of an acceptable scientific standard, however I have significant reservations, as outlined above.

# Response

**Published:** 2018-06-19

**Authors:** Abbas Mansour

**Affiliations:** Faiha Specialized Diabetes, Endocrine and Metabolism Center (FDEMC), Iraq

The study by Mansour AA et al. is an important contribution to the limited pituitary research in the middle East and North Africa (MENA) region.

The following points need further modifications/clarifications;

- Sellar and parasellar region lesions usually refers to structural abnormalities and do not necessarily include other pituitary pathologies like hyperprolactinemia and hypopituitarism. Therefore, I suggest using the term ''pituitary disorders'' instead; more comprehensive.

Done

 

- The inclusion of patients needs further clarifications in the ''Method'' section. what diagnoses where looked for?

Retrospective data analysis of FDEMC  database for the period from January 2012 through June 2017.

**Inclusion criteria**:We included all patients with pituitary disorders who have MRI pitutiary regardles the age .

 

- Pituitary adenoma includes all functioning and non-functioning pituitary tumors. In some part of the text, it was used separately from its subtype (see paragraph; Definition of variables). Maybe just typo.

  Done

- Table (2) needs to be simplified. The total number of patients should equal clearly equal 232. I suggest using 3-4 categories only; Sellar masses/abnormalities, Hyperprolactinemia, hypopituitarism and others.  

Its cant be 232 because some may have more than one diagnosis

 

- The "miscellaneous" cases need to be mentioned; what diagnoses are here?

Miscellaneous includes galactorrhea, hypogonadotropic hypogonadism, and acromegaloidism

 

- Table 3 the sum of patient with pituitary adenoma subtypes are not equal to the  total number of cases.

GH-secreting adenoma,2 of them stain on biopsy for lactotroph cell

GH-secreting adenoma with hyperprolactinemia in 5 patients

 

- In the conclusion, ''A change in the practice of adenoma treatment is needed'' is very general. Based on the presented data, change in the management of GH secreting adenomas is more specific and relevant.

A change in the practice of pituitary adenoma treatment is needed.

All adenomas treatment needed reevaluation .

 

- Additional language editing will further enhance the manuscript.

Done

# Peer review: Referee response for version 1

**DOI:** 10.5256/f1000research.14810.r32887

**Published:** 2018-04-30

**Authors:** Abdul Al-Toma

**Affiliations:** 1 Department of Internal Medicine, Gastroenterology and Hepatology, St Antonius Hospital, Nieuwegein, Netherlands

Dr. Mansour and co-authors investigated the Spectrum of pituitary disease (Sellar and Parasellar Region Lesions) in a large cohort of patients from a tertiary referral center in the southern region of Iraq. This center receives patients with pituitary diseases from most of Southern Iraq, a population of 6-8 million.

 The authors provided a well written retrospective analysis of the study population. The manuscript is focused on providing an attractive epidemiological description of the studied patients.  

**Abstract section**: concise text and states clearly the objective of the report. In their conclusion they stated that a change in practice of adenoma treatment is needed. It would be more informative if they provide some data on how they reached to this conclusion. A minor correction: the following 2 sentences need to be separated by full stop mark instead of comma ( *Pituitary adenoma constituted the bulk of sellar and parasellar region lesions, growth hormone secreting adenoma is the most common adenoma followed by NFPA and prolactinoma due to referral bias*.)

 The following sections are well written and data were clearly presented:

**Methods and results**. In addition they included an appropriate Statistical analysis. However, minor English language edition is needed.

**The discussion section** described the study results in comparison with recent literature. Recent literature has been adequately addressed. The authors stated the limitation f their manuscript clearly. It would be interesting to provide some information on the prognosis of their study patients, both for surgically or medically treated patients.

**The references** are clearly presented and conform the current standards.

**The tables** are clearly written.

**Minor comments**: although the manuscript is well written, however some revision of the English text needs to done.

I have read this submission. I believe that I have an appropriate level of expertise to confirm that it is of an acceptable scientific standard.
